# Quantitative Analysis of Forest Fragmentation in the Atlantic Forest Reveals More Threatened Bird Species than the Current Red List

**DOI:** 10.1371/journal.pone.0065357

**Published:** 2013-05-29

**Authors:** Jessica K. Schnell, Grant M. Harris, Stuart L. Pimm, Gareth J. Russell

**Affiliations:** 1 Department of Biological Sciences, Rutgers University, Newark, New Jersey, United States of America; 2 Department of Biological Sciences, New Jersey Institute of Technology, Newark, New Jersey, United States of America; 3 United States Fish and Wildlife Service, Albuquerque, New Mexico, United States of America; 4 Nicholas School of the Environment, Duke University, Durham, North Carolina, United States of America; University of Alberta, Canada

## Abstract

Habitat loss and attendant fragmentation threaten the existence of many species. Conserving these species requires a straightforward and objective method that quantifies how these factors affect their survival. Therefore, we compared a variety of metrics that assess habitat fragmentation in bird ranges, using the geographical ranges of 127 forest endemic passerine birds inhabiting the Atlantic Forest of Brazil. A common, non-biological metric — cumulative area of size-ranked fragments within a species range — was misleading, as the least threatened species had the most habitat fragmentation. Instead, we recommend a modified version of *metapopulation capacity*. The metric links detailed spatial information on fragment sizes and spatial configuration to the birds’ abilities to occupy and disperse across large areas (100,000+ km^2^). In the Atlantic Forest, metapopulation capacities were largely bimodal, in that most species’ ranges had either low capacity (high risk of extinction) or high capacity (very small risk of extinction). This pattern persisted within taxonomically and ecologically homogenous groups, indicating that it is driven by fragmentation patterns and not differences in species ecology. Worryingly, we found IUCN considers some 28 of 58 species in the low metapopulation capacity cluster to not be threatened. We propose that assessing the effect of fragmentation will separate species more clearly into distinct risk categories than does a simple assessment of remaining habitat.

## Introduction

Assessing a species’ risk of extinction is a core activity for conservation science. We must identify the species that need protection and then consider how to provide it. Moreover, individual species’ assessments provide the elements to set priorities for areas that may differ greatly in how many threatened species they contain. The International Union for Conservation of Nature (IUCN) assesses threat for species globally. IUCN’s scheme groups species deemed threatened into three main classes: Critically Endangered (CR), Endangered (EN), Vulnerable (VU) and two non-threatened classes: Near Threatened (NT) and Least Concern (LC). These categories rely on well-defined criteria. For birds, IUCN delegates assessments to BirdLife International, which in turn recruits thousands of individuals to contribute to species’ assessments. Our experiences in helping such assessments motivate our seeking more consistent and reliable measures of risk that employ readily available data to refine geographical ranges.

To date, we have used elevation and land-cover data to trim the BirdLife range maps to produce more realistic ones [1]. In doing so, we observed that some ranges are much smaller than previously thought and some are massively fragmented. We now seek to advance this aspect of species’ assessments towards an even more consistent, quantitative framework. Until recently, that has been hard to implement broadly. In this paper, we compare a number of methods of quantifying habitat fragmentation. Our worrying conclusion is that some species are likely more threatened than currently expected.

Habitat loss exterminates species [2], and threatens many more [3]–[4]. The species BirdLife deems threatened are overwhelmingly those with currently small geographical ranges [5]. Indeed, for the terrestrial species we consider here, two factors dominate: some measure of declining population numbers — most often assessed indirectly by continuing habitat loss — and a small geographical range. Thus, range size is an explicit criterion and is sufficient, though not necessary, to give threatened status. From a conservation standpoint, we must estimate range size appropriately. The need is particularly acute for species in montane areas, where the ranges that fall within known elevation limits may be very much smaller than those shown by BirdLife maps [1].

Habitat fragmentation compounds this problem [6]–[8]. Habitat fragmentation, and its relevance towards extinction [8]–[12], has been studied exhaustively in birds [4], [13]–[35]. The issue is well understood theoretically: as population size increases, the risk of stochastic extinction drops precipitously. Empirical data for birds on real islands and forest “islands” surrounded by agricultural land readily confirm the theory [14], [30], [36]–[37]. Thus, two species with identical range sizes will differ sharply in risk if one range is composed of continuous habitat, while the other exists in tiny fragments. Using species loss curves that record bird extinction from forest fragments, for example, one study [30] recommended that individual forest fragments be a minimum of 10 km^2^ for long-term within-patch survival.

To simplify, our process to generate objective and reliable threat assessments relies on the size and fragmentation of a bird’s range. It has three stages:

1. Existing IUCN criteria use what we can think of as “field guide ranges” — technically Extent of Occupancy estimates [38]. These are maps with generally smooth boundaries. They do not factor in realistic habitat requirements except in general terms. The ranges are typically continuous, though there could be a few isolated populations. Employing these maps, IUCN sets a threshold of 20,000 km^2^ below which a species is likely to be threatened, given assumed continuing loss of habitat or population.

2. Unsatisfied with how this applied to terrestrial bird species, Harris and Pimm [1] trimmed those ranges by elevation, broadly suitable habitat, and remaining forest. These maps showed inevitably smaller areas that typically had convoluted boundaries. Those for montane species followed contour lines, for example, even in regions of intact forest. The authors suggested that a threshold of 11,000 km^2^, below which a species is at particular risk, would ensure consistency in listing. In doing so, they added to the list of putatively threatened species those that had very much smaller ranges than their Extent of Occupancy estimates. Note, these maps still show the extent of suitable habitat, or *potential* range, rather than occupied range. Henceforward, for simplicity, we will refer to this simply as ‘range.’ We know that any given species is unlikely to occupy all the available habitat, especially when it exists in tiny fragments. As will become clear, we build that effect into our analysis.

3. For most species, these trimmed maps uncovered highly fragmented ranges. To derive their threshold, Harris and Pimm [1] assumed that the fragmentation was broadly comparable across species. By inspection, that assumption is invalid. Some species have ranges that are larger than 11,000 km^2^, yet heavily fragmented. The IUCN acknowledges that fragmentation is a distinct problem for species, separate from the problem of small ranges alone [38]. They also have a history of updating criteria to be more consistently quantitative [39]–[41], especially Criterion E “quantitative analysis of extinction probability” (http://www.iucnredlist.org/static/categories_criteria_3_1, [42]). Still, there is no accepted, standardized method that quantifies range fragmentation and links it to extinction threat for that species. Providing one is this paper’s objective.

There have been many previous attempts to quantify fragmentation. The FRAGSTATS program exemplifies ‘spatial-only’ metrics, and can produce area, edge, shape, and nearest neighbor metrics, among other things [43]. As an example of a spatial-only metric, we examine plots describing the cumulative amount of area covered by size-ranked fragments within a bird’s range. As we will describe below, this approach can mislead. Moreover, such approaches do not relate directly to extinction risk.

Among the most complex kinds of approach, METAPHOR relies on a discrete-time, stochastic individual-based model that simulates landscape effects on metapopulation persistence [44]–[48]. Relatedly, one can derive ecologically scaled landscape indices (ESLI) from landscapes and account for dispersal and carrying capacity [47]–[48]. However, individual-based movement models typically require many parameters, describing both the landscape and the behavioral choices made by individuals. Many of them are likely to be poorly known and difficult to estimate.

Our method treads a middle ground by adapting basic metapopulation theory. Many studies have pioneered and employed metapopulation dynamics on butterflies, mammals, plants, and plant-herbivore-parasitoid communities (reviews in [49]–[50]) to examine patterns of extinction and colonization. Despite their being highly informative, these approaches have generally not involved large spatial scales and, indeed have a critical failing in this regard. Our adaptation [51] addresses this deficit by a simple modification to the spatially explicit metapopulation model that allows it to describe species’ abilities to occupy and disperse among fragments across a landscape covering thousands of square kilometers or more.

Following Schnell et al. [51], we employ a modified version of metapopulation capacity [52] as the framework for quantifying fragmentation to inform threat assessments. Metapopulation capacity measures the contribution of the spatial extent configuration of a landscape of habitat patches to the long-term persistence of a species living in those patches. The metric incorporates information on a species’ ability to disperse and on patch areas and their separation. In this regard, it forms a standardized way to incorporate fragmentation in Criterion E. As we will show, it also identifies potential errors in current threat assessments for birds in the Atlantic Forest, and by doing so demonstrates how threat assessments can be improved to generate greater consistency.

## Materials and Methods

### Range data

Harris and Pimm [1] collected field guide ranges, elevation, and forest ecotype data, and overlaid them to generate historical range estimates. These predictions were then further refined with satellite images of forest cover to produce current range estimates. Of their four study sites, we focus here on the Atlantic Forest of Brazil.

The Atlantic forest of southeastern Brazil is a biodiversity hotspot [53], with high levels of endemism, only 6–8% of forest remaining, and extensive fragmentation [54]–[56]. Birds with small ranges are often in areas where there are higher than expected numbers of threatened species. The Atlantic forest stands out, with endemics being particularly threatened, unable to withstand the forest fragmentation [5], [57]–[59]. The study of this site is particularly important in quantifying fragmentation effects for many bird species in peril because it is a prime example of an endemic bird area (EBA) and a site where both extreme fragmentation and many threatened bird species occur [5], [53]. Forest maps of this region, at a 1 km^2^ scale, form the basis of all the metrics described below.

The Atlantic forest of southeastern Brazil is a biodiversity hotspot [53], with high levels of endemism, only 6–8% of forest remaining, extensive fragmentation, and exceptional numbers of threatened species [54]–[56]. Forest maps of this region, at a 1 km^2^ scale, form the basis of all the metrics described below.

### Range size

Range size is the simplest metric, ignoring fragmentation completely, and the metric Harris and Pimm [1] used. The area estimates are usually much smaller than the ‘extent of occurrence’ area values cited in BirdLife evaluations. (EOO is more similar to the ‘field-guide’ range.)

### Forest fragment cumulative area distributions

One method of assessing fragmentation is to examine the distribution of sizes of forest fragments. A standard technique plots the cumulative total area contained in patches below a certain area, against that area [60]–[62]. On a log-log scale, this plot is typically approximately linear over a very wide range of areas, and contains much useful information. The right-most point yields both the size of the largest fragment (its *x*-axis value) and the total area of all the fragments (its *y*-axis value). The slope describes the fraction of total area contained in progressively smaller fragments — the shallower the slope, the more the system is fragmented (but see Results). These metrics takes no account of the spatial separation or distribution of patches.

### Metapopulation-based metrics

Taking spatially explicit metapopulation models [49], [63]–[64] as a starting point (See Appendix 1), we previously proposed two metrics of fragmentation. One is a simple modification of metapopulation capacity, which is a measure of how the spatial configuration of a set of patches contributes to estimates of long-term metapopulation persistence [52]. Our modification allowed self-colonization, a biologically sensible phenomenon when considering large patches; larger patches are more likely to retain survivors in the event of an extinction threat to that patch, allowing a ‘rescue effect’ to occur within the patch itself. We obtain our modified metapopulation capacity (λ*_self_*) by taking the leading eigenvalue of the matrix *M* with elements
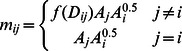



where *A_i_* is the area of the *i*th patch, *x* is an exponent that scales extinction probability to area, and *f*(*D_ij_*) is a dispersal function describing how arrival rate drops off with the distance *D_ij_* between two patches *i* and *j*. Here, *D_ij_* is the minimum edge-to-edge distance between patches, and for *f*(*D_ij_*) we used a survival-rate transformation of the log-sech dispersal kernel proposed by Van Houtan et al. [65] for forest birds in the Amazon (See Methods S1 for more details on the derivation of modified metapopulation capacity and the log-sech survival function).

Metapopulation capacity is analogous to the concept of effective population size in population genetics, and its units can be thought of as “Levins patch equivalents”, meaning the number of patches in an equivalently-behaving, non-spatially-explicit metapopulation [51].

### Threat status

We used a slightly more updated version of BirdLife threat designations [66] than Harris & Pimm did [1]. Using the updated threats resulted in one species removed from our analysis for having only one 1 km^2^ patch remaining in its range (*Philydor novaes*i – CR) and another for being extinct in the wild (*Mitu mitu* – EW), as well as three status changes.

## Results

We calculated range area (trimmed by elevation and forest cover), the cumulative area of fragments within a bird’s range, and the two metapopulation measures, for 127 passerine forest birds of the Atlantic Forest of Brazil. This area has the largest concentration of threatened bird species in the Americas [5].

### Cumulative patch area plots

**Figure 1:**
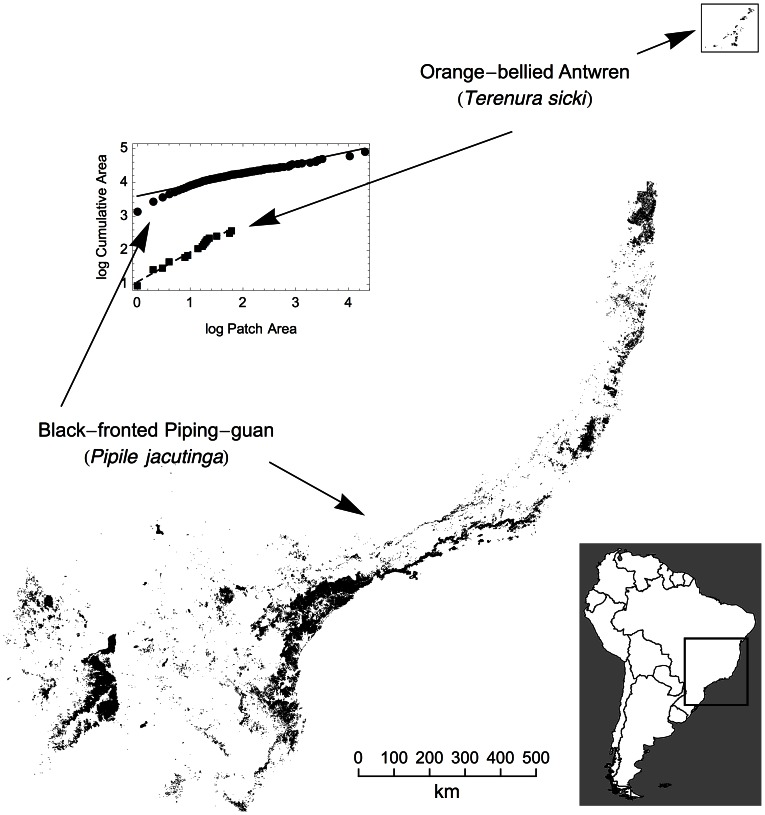
Cumulative area plots of two Atlantic forest birds, the large-ranged black-fronted piping-guan (*Pipile jacutinga*) and the small-ranged orange-bellied antwren (*Tenenura sicki* — highlighted with a box). Graph axes are the logarithm of cumulative area in patches up to a given size, versus the logarithm of that patch size. The lines are fitted linear regressions. A steep (more positive) slope, e.g. orange-bellied antwren, indicates that a range is composed mainly of relatively larger patches. Shallow slopes, e.g. black-fronted piping-guan, indicate birds with ranges composed of relatively many smaller forest fragments. Counter-intuitively, while the guan has a larger overall range and larger patches within that range, the shallower slope of its plot suggests that it is more fragmented (see text for further discussion).

Though easy to understand, these plots can seriously mislead. Consider two Endangered species as examples: the black-fronted piping-guan (*Pipile jacutinga*) has a much larger total area and maximum patch size than the orange-bellied antwren (*Terenura sicki*) (Fig. 1). However, the slope of its cumulative area plot is shallower, indicating that a greater fraction of its range is made up of smaller patches. Conventional wisdom would interpret this as greater fragmentation, and in a sense that is correct. Species with larger overall range typically have a small number of larger patches, surrounded by a constellation of (sometimes hundreds of) smaller patches. These small patches raise the intercept and reduce the slope of the cumulative area plot.

The problem is that this spatial pattern fails to capture the ecological effects of fragmentation. Well-understood ecology tells us that most of the tiny patches will usually be unoccupied, and therefore contribute almost nothing to species’ persistence. The sizes and spatial relationships of the smaller number of large patches determine almost all of the landscape’s ability to support that species. In any sensible metric, the existence of many small patches around some large patches should not make the situation seem worse than if the small patches were not present at all. Unfortunately, that is what the cumulative area plot does. Furthermore, it ignores patch spacing and configuration, two factors vitally important to dispersing organisms.

There is a second, practical problem with the cumulative area approach. Landscape data typically come at a certain fixed resolution that determines the smallest possible size of a patch (one pixel). If the smallest range species (e.g. orange-bellied antwren seen here) occupy only a few pixels overall, then there is a constraint on how fragmented that range can appear because the largest patch cannot be much bigger than the smallest. (Were we to use a smaller pixel size, the small-range species might seem more fragmented, but the effect would hold true for large-range species, so it would not matter in a relative sense.)

We conclude that using habitat fragmentation to assess species threat requires us to incorporate ecology, in the form of species’ abilities to occupy and disperse between fragments.

### Range area and metapopulation capacity

Some 30 out of 127 passerine species fall below Harris & Pimm’s proposed threshold describing extinction threat – 11,000 km^2^ [1]. Of these birds, Birdlife does not list seven (23%) in the three classes that constitute “threatened.” Of particular concern are the three species that the BirdLife lists as Least Concern: Serra Do Mar tyrant-manakin (*Neopelma chrysolophum*), minute hermit (*Phaethornis idaliae*), and white-bibbed antbird (*Myrmeciza loricata*).

**Figure 2:**
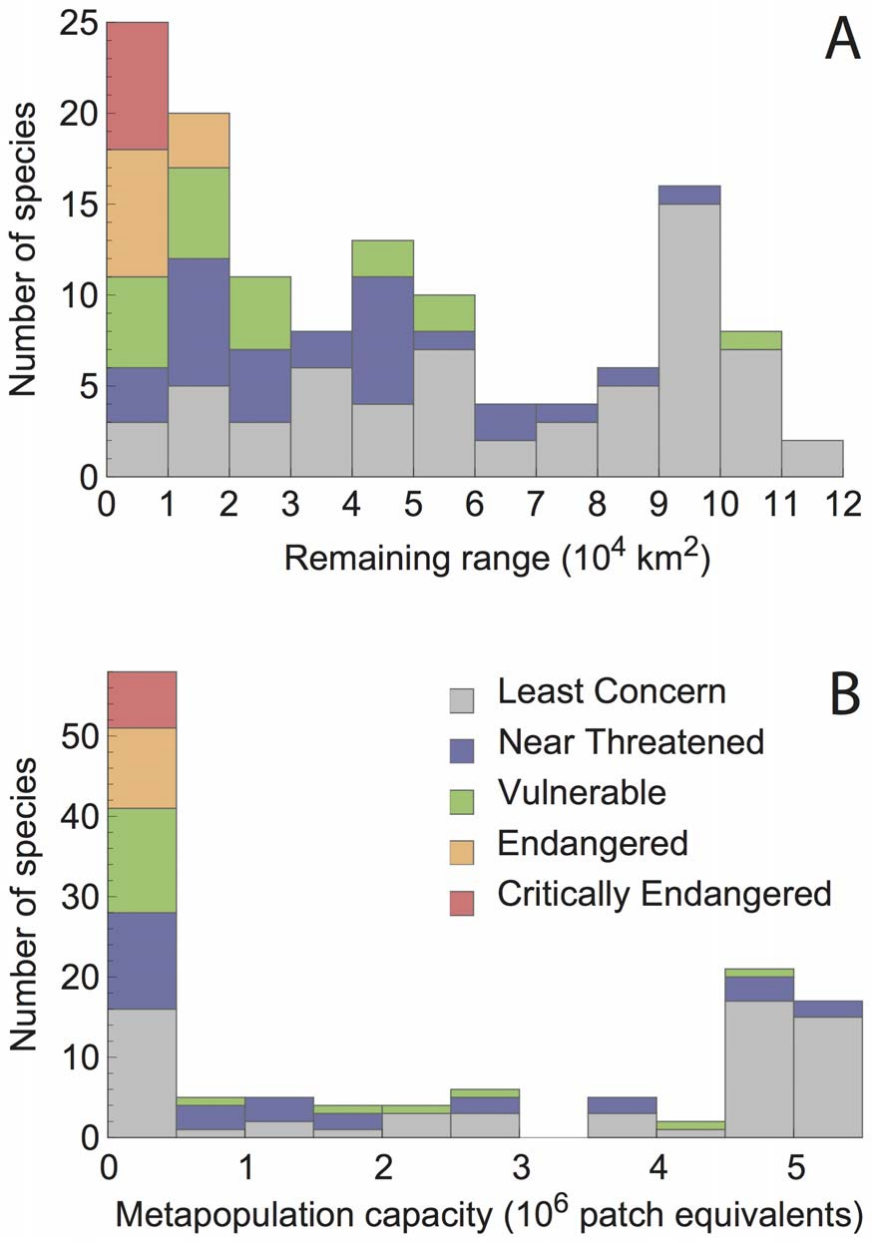
For 127 passerine birds of the Atlantic forest of Brazil, differences are evident between two measures of extinction risk: A) the area of a bird’s remaining range; B) metapopulation capacity, which accounts for range fragmentation as well as area. Colors indicate IUCN Red List categories with red, orange, and green being the three ‘threatened’ categories. When accounting for the long-term extinction risk associated with fragmentation, a distinct group of 57 species with very low metapopulation capacity becomes apparent, of which 28 are deemed non-threatened by BirdLife.

We used histograms to present and compare alternative metrics for assessing extinction risk (Fig. 2). Across passerines, the remaining range area shows a range of values (Fig. 2A). As expected, species listed as threatened are more often in the small-range categories. By comparison, values for metapopulation capacity (Fig. 2B) are distinctly bimodal, as species cluster at either end of the metric’s range. Examination of the birds’ range maps reveals, unsurprisingly, that whether the range includes one or more large forest patches determines the difference between a high and low value for metapopulation capacity. This accords with ecological theory and data (see Introduction).

The 58 species in the smallest metapopulation capacity class include 31 (84%) of the 37 BirdLife threatened species. They also include 28 species not threatened (shaded blue or grey), yet whose range fragmentation is very similar. This is a potential omission rate of 48% — double that of the simple analysis of area.

**Figure 3:**
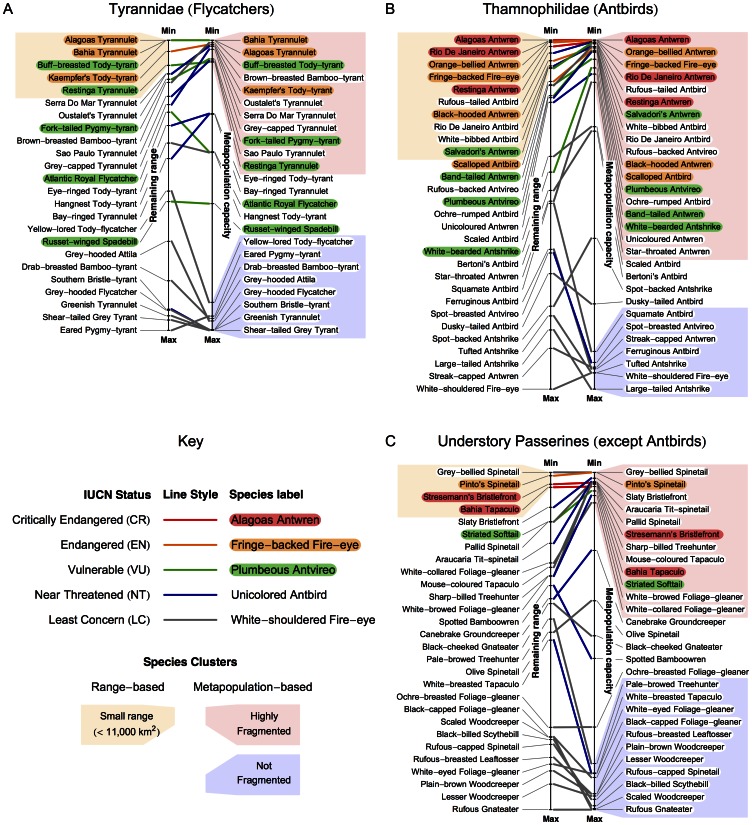
Slopegraphs (ref. [69]) comparing relative values of remaining range (left axis) and modified metapopulation capacity (right axis) for 2 subsets of bird species endemic to Brazil’s Atlantic forest. Thick, center lines connect the value of remaining range to the value of metapopulation capacity for the same species. Thinner, gray lines on either side link to species labels, which are spread out for clarity. The colors of the center lines and the species’ label backgrounds reflect IUCN status (see key); only the names of species in the three threatened categories are colored. Clusters of species are highlighted, to indicate those of low range area, and high and low extinction risk due to fragmentation.

Perhaps BirdLife considers differences in secondary habitat tolerance or dispersal ability in making their rankings. Previously, we found that secondary habitat use did not affect rankings when compared to remaining area of primary habitat [67]. Moreover, if basic requirements (i.e., primary habitat) are inadequate, mobility of forest-dependent birds is unlikely to compensate [68]. Nevertheless, to evaluate these effects we separated out three groups of species by increasing taxonomic and ecological similarity. We reapplied our metapopulation capacity analysis separately for each group. Since fewer species are in each group, we present results with a slopegraph [69]. These slopegraphs compare the relative values of species’ remaining ranges (the metric of [2]) on the left side, and metapopulation capacities on the right side, with birds at greater extinction risk (smaller values) at the top and those at lesser risk (larger values) at the bottom (Fig. 3).

The two family groups consisted of tyrant flycatchers (family Tyrannidae, Fig. 3A) and antbirds (family Thamnophilidae, Fig. 3B). These taxa are morphologically and ecologically homogenous, yet a clear bimodal distribution of extinction risk is evident. Five and seven species in each taxon respectively occur in an obvious ‘low capacity’ cluster (pink background), yet BirdLife do not consider them to be threatened (respective omission rates of 45% and 39%). These results virtually eliminate the objection that the BirdLife omissions stem from differences in secondary habitat tolerance or species dispersal abilities.

For the Tyrannidae, the high-risk species [66] BirdLife overlook include brown-breasted bamboo-tyrant (*Hemitriccus obsoletus*), Oustalet's tyrannulet (*Phylloscartes oustaleti*), Serra Do Mar tyrannulet (*Phylloscartes difficilis*), grey-capped tyrannulet (*Phyllomyias griseocapilla*), and São Paulo tyrannulet (*Phylloscartes paulistus*). All species except the brown-breasted bamboo-tyrant (LC) are Near Threatened due to suspected rapid declines from habitat loss. All except the São Paulo tyrannulet have no quantified population size. Their local abundances range from “uncommon” to “fairly common.” All species except the grey-capped tyrannulet (medium dependency) have “high forest dependency”. All share the habitat of subtropical/tropical moist forest, either lowland or montane.

For the Thamnophilidae, the possible misclassified species include rufous-tailed antbird (*Drymophila genei*), white-bibbed antbird (*Myrmeciza loricata*), Rio de Janeiro antbird (*Cercomacra brasiliana*), rufous-backed antvireo (*Dysithamnus xanthopterus*), ochre-rumped antbird (*Drymophila ochropyga*), unicoloured antwren (*Myrmotherula unicolor*), and star-throated antwren (*Myrmotherula gularis*). All species are Least Concern, since their numbers are suspected to be either stable or in decline, and any decline that might be happening is not rapid enough to warrant threatened status. All species except the unicoloured antwren have no quantified population size, and vary from “common” to “rare”. All species have “high forest dependency” except the Rio de Janeiro antbird (medium dependency). All live in some type of subtropical/tropical forest.

The remaining “understory specialists” (Fig. 3C) included Conopophagidae, Dendrocolaptidae, Furnariidae, and Rhinocryptidae. These species have similar body sizes and likely similar dispersal abilities. The bimodality of the metapopulation capacity metric remains clearly visible. The cluster of 12 low-capacity ranges at the top of this list includes eight species (50%) that BirdLife does not consider threatened grey-bellied spinetail (Synallaxis cinerascens*), slaty bristlefront (Merulaxis ater*), Aracuaria tit-spinetail (Leptasthenura setaria*), pallid spinetail (Cranioleuca pallida*), sharp-billed treehunter (Heliobletus contaminates*), mouse-coloured tapaculo (Scytalopus speluncae*), white-browed foliage-gleaner (*Anabacerthia amaurotis*) and white-collared foliage-gleaner (*Anabazenops fuscus*). The five of these marked with an asterisk have smaller capacities than the Bahia tapaculo (*Eleoscytalopus psychopompus*), a species listed as Critically Endangered. These species are also candidates for immediate reexamination.

We note that some species fall into a ‘high capacity’ cluster (pale blue background). These species have a combination of a large overall range and a number of large contiguous patches within that range (see, for example, the black-fronted piping-guan in Fig. 1). We also list the species in the ‘low capacity’ cluster when considering all our species’ ranges from the Atlantic forest (Table S1).

## Discussion

Modified metapopulation capacity is an objective and consistent metric for assessing the effect of fragmentation on extinction risk. For a large sample of birds, most species fell into either a low capacity (high risk) cluster or a high capacity (low risk) cluster, and this bimodal pattern remained consistent within taxonomically and ecologically homogenous groups. Therefore, the fragmentation patterns in the Atlantic Forest determine it, not the differences between the varying natural histories of these species. The bimodality makes the process of including habitat fragmentation in overall threat assessments relatively simple and straightforward.

High metapopulation capacity clearly links to the existence of substantial patches of remaining habitat, in which we expect large sub-populations to persist for long periods. There is, therefore, every reason to expect that the patterns we describe should apply to other taxa and other regions.

Because metapopulation capacity is a relative measure [64], it will be most useful as a ‘peer group’ comparison. That is the way we have used it here. As one completes the risk assessment process for more species, this type of comparison becomes straightforward. One can base peer groups on phylogeny/taxonomy, or any sensible combination of morphology and ecology (traits of which tend to follow phylogeny — [70]). Specific life history characteristics can make some groups, such as ant-followers [71], or ground nesters ([72], etc.), particularly vulnerable to extinction [73]. This is likely due in part to the influence of these characteristics on dispersal behavior. Metapopulation capacity can help distinguish those within each group that are most at risk.

Ideally, these measurements would improve with real values of dispersal traits. Combined with existing extinction rate estimates, these would allow us to say whether a given fragmented range is capable of supporting a species or not. Unfortunately, dispersal habits remain unknown for most species. They are challenging to measure, requiring the tracking of many small and vagile animals in remote and inaccessible locations. Even when inferences can be made indirectly, such as from presence/absence snapshots from patch systems, or with repeated surveys [74]–[75], the necessary data remain sparse, and the parameters frequently uncertain. More generally, we need a better understanding of animal movements in complex landscapes, as straight-line distances are unlikely to be a good model of relative movement rates for many species. For now, correlations between known life history characteristics and dispersal traits may allow us to estimate values themselves, rather than just delineating peer groups.

Fortunately, some information can guide us. For example, dispersal distances of avian species correlate with body size and breeding territory size [76], [77]. Understory birds are thought of as less able dispersers in fragmented habitat, but this is an over-generalization [78]–[80]. For instance, Van Houtan et al. [65] found that persisting species moved *less* than extinction-prone species in intact forest, but moved further after fragmentation. Hansbauer et al. [78] found that some understory species showed no change in movement with the occurrence of fragmentation, while others increased speed and the distances traveled. Hansbauer et al. [79] also found that one understory species increased its range to include matrix habitat, while another (an army ant follower) did so only at food source locations, while a third never ventured outside of intact forest.

Given the potential for improved understanding of dispersal, a key advantage of the metapopulation approach is that it is flexible and adaptable, allowing us to incorporate new data and even dispersal models as they become available. We recommend it as a framework to the IUCN and other organizations involved in species risk assessment.

We understand from our own experiences in species assessments that the process for assessing threat is long and complex. That said, what the results of Harris and Pimm [1] and those herein suggest is a list of candidate species for which existing decisions of non-threatened status may be in serious error. We urge that those who assess species should examine candidates even more carefully before excluding them from the lists of species for which we should have special concern.

## Supporting Information

Methods S1 Spatially explicit metapopulation models and metapopulation capacity.
(DOCX)

Table S1 Complete list of Atlantic forest bird species (bold names recommended for reassessment based on habitat fragmentation, due to low metapopulation capacity).
(XLSX)

## References

[1] Harris GM, Pimm SL (2008) Range size and extinction risk in forest birds. Conserv Biol 22: 163–171. 18254861 10.1111/j.1523-1739.2007.00798.x

[2] Pimm SL, Askins RA (1995) Forest losses predict bird extinctions in eastern North America. Proc Natl Acad Sci USA 92: 9343–9347. 11607581 10.1073/pnas.92.20.9343 PMC40981

[3] Brooks TM, Pimm SL, Collar NJ (1997) Deforestation predicts the number of threatened birds in insular southeast Asia. Conserv Biol 11: 382–384.

[4] Brooks TM, Tobias J, Balmford A (1999) Deforestation and bird extinctions in the Atlantic forest. Anim Conserv 2: 211–222.

[5] Manne LL, Brooks TM, Pimm SL (1999) Relative risk of extinction of passerine birds on continents and islands. Nature 399: 258–261.

[6] Saunders DA, Hobbs RJ, Margules CR (1991) Biological consequences of ecosystem fragmentation: a review. Conserv Biol 5: 18–32.

[7] Franklin AB, Noon BR, George TL (2002) What is habitat fragmentation? Stud Avian Biol 25: 20–29.

[8] Fahrig L (2003) Effects of habitat fragmentation on biodiversity. Annu Rev Ecol Syst 34: 487–515.

[9] Debinski DM, Holt RD (2000) Review: A survey and overview of habitat fragmentation experiments. Conserv Biol 14: 342–355.

[10] Laurance WF, Lovejoy TE, Vasconcelos HL, Bruna EM, Didham RK, et al (2002) Ecosystem decay of Amazonian forest fragments: a 22-Year investigation. Conserv Biol 16: 605–618.

[11] Laurance WF, Camargo JL, Luizão RC, Laurance SG, Pimm SL, et al (2011) The fate of Amazonian forest fragments: a 32-year investigation. Biol Conserv 144: 56–67.

[12] Henle K, Davies KF, Kleyer M, Margules C, Settele J (2004) Predictors of species sensitivity to fragmentation. Biodivers Conserv 13: 207–251.

[13] Galli AE, Leck CF, Forman RTT (1976) Avian distribution patterns in forest islands of different sizes in central New Jersey. Auk 93: 356–364.

[14] Pimm SL, Jones HL, Diamond J (1988) On the risk of extinction. Am Nat 132: 757–785.

[15] Bierregaard Jr RO, Lovejoy TE (1989) Effects of forest fragmentation on Amazonian understory bird communities. Acta Amazon 19: 215–241.

[16] Harper LH (1989) The persistence of ant-following birds in small Amazonian forest fragments. Acta Amazon 19: 249–263.

[17] Vuilleumier F (1970) Insular biogeography in continental regions. I. The northern Andes of South America. Am Nat 104: 373–388.

[18] Newmark WD (1991) Tropical forest fragmentation and the local extinction of understory birds in the eastern Usambara Mountains, Tanzania. Conserv Biol 5: 67–78.

[19] Bierregaard Jr RO, Lovejoy TE, Kapos V, Augusto A, Hutchings RW (1992) The biological dynamics of tropical rainforest fragments. BioScience 42: 859–866.

[20] Estrada A, Coates-Estrada R, Meritt D, Montiel S, Curiel D (1993) Patterns of frugivore species richness and abundance in forest islands and in agricultural habitats at Los Tuxtlas, Mexico. Plant Ecol 107–108: 245–257.

[21] Maurer BA, Heywood SG (1993) Geographic range fragmentation and abundance in neotropical migratory birds. Conserv Biol 7: 501–509.

[22] Andrén H (1994) Effects of habitat fragmentation on birds and mammals in landscapes with different proportions of suitable habitat: a review. Oikos 71: 355–366.

[23] Stouffer PC, Bierregaard Jr RO (1995) Use of Amazonian forest fragments by understory insectivorous birds. Ecology 76: 2429–2445.

[24] Hagan JM, Matthew Vander Haegen W, McKinley PS (1996) The early development of forest fragmentation effects on birds. Conserv Biol 10: 188–202.

[25] Hinsley SA, Pakeman R, Bellamy PE, Newton I (1996) Influences of habitat fragmentation on bird species distributions and regional population sizes. Proc Roy Soc Lond B Bio 263: 307–313.

[26] Turner IM (1996) Species loss in fragments of tropical rain forest: a review of the evidence. J Appl Ecol 33: 200–209.

[27] Gascon C, Lovejoy TE, Bierregaard Jr RO, Malcolm JR, Stouffer PC, et al (1999) Matrix habitat and species richness in tropical forest remnants. Biol Conserv 91: 223–229.

[28] Stratford JA, Stouffer PC (1999) Local extinctions of terrestrial insectivorous birds in a fragmented landscape near Manaus, Brazil. Conserv Biol 13: 1416–1423.

[29] Crooks KR, Suarez AV, Bolger DT, Soulé ME (2001) Extinction and colonization of birds on habitat islands. Conserv Biol 15: 159–172.

[30] Ferraz G, Russell GJ, Stouffer PC, Bierregaard RO Jr, Pimm SL, et al (2003) Rates of species loss from Amazonian forest fragments. Proc Natl Acad Sci USA 100: 14069–14073. 14614134 10.1073/pnas.2336195100 PMC283547

[31] Antongiovanni M, Metzger JP (2005) Influence of matrix habitats on the occurrence of insectivorous bird species in Amazonian forest fragments. Biol Conserv 122: 441–451.

[32] Borgella R, Gavin TA (2005) Avian community dynamics in a fragmented tropical landscape. Ecol Appl 15: 1062–1073.

[33] Stouffer PC, Bierregaard Jr RO, Strong C, Lovejoy TE (2006) Long-term landscape change and bird abundance in Amazonian rainforest fragments. Conserv Biol 20: 1212–1223. 16922237 10.1111/j.1523-1739.2006.00427.x

[34] Feeley KJ, Gillespie TW, Lebbin DJ, Walter HS (2007) Species characteristics associated with extinction vulnerability and nestedness rankings of birds in tropical forest fragments. Anim Conserv 10: 493–501.

[35] Stouffer PC, Strong C, Naka LN (2009) Twenty years of understorey bird extinctions from Amazonian rain forest fragments: consistent trends and landscape-mediated dynamics. Divers Distrib 15: 88–97.

[36] Pimm SL, Diamond J, Reed TM, Russell GJ, Verner J (1993) Times to extinction for small populations of large birds. Proc Natl Acad Sci USA 90: 10871–10875. 11607439 10.1073/pnas.90.22.10871 PMC47880

[37] Russell GJ, Diamond JM, Reed TM, Pimm SL (2006) Breeding birds on small islands: island biogeography or optimal foraging? J Anim Ecol 75: 324–339. 16637986 10.1111/j.1365-2656.2006.01052.x

[38] IUCN (2001) IUCN Red List Categories and Criteria: Version 3.1. IUCN Species Survival Commission. IUCN: Gland, Switzerland, and Cambridge, UK.

[39] Mace GM, Lande R (1991) Assessing extinction threats: toward a reevaluation of IUCN threatened species categories. Conserv Biol 5: 148–157.

[40] Mace GM (2008) Quantification of extinction risk: IUCN’s system for classifying threatened species. Conserv Biol 22: 1424–1442. 18847444 10.1111/j.1523-1739.2008.01044.x

[41] Butchart SHM, Akçakaya HR, Chanson J, Baillie JEM, Collen B, et al (2007) Improvements to the Red List Index. PLoS ONE 2: e140. 17206275 10.1371/journal.pone.0000140 PMC1764037

[42] Brooke M de L (2009) A necessary adjustment of the extinction risk associated with the red list criteria? Avian Conserv Ecol 4: 1.

[43] McGarigal K, Marks BJ (1995) FRAGSTATS: spatial pattern analysis program for quantifying landscape structure. Gen. Tech. Rep. PNW-GTR-351. Portland, OR: U.S. Department of Agriculture, Forest Service, Pacific Northwest Research Station. 122 p.

[44] Verboom J (1996) Modelling fragmented populations: between theory and application in landscape planning. Ph.D. thesis. Scientific contribution no. 3 of Institute for Forestry and Nature Research (IBN-DLO), Wageningen, The Netherlands.

[45] Foppen RPB, ter Braak CFT, Verboom J, Reijnen R (1999) Sedge Warblers (*Acrocephalus schoenobaenus*) and African rainfall, a low population resilience in fragmented marshlands. Ardea 87: 113–127.

[46] Foppen RPB, Chardon JP, Liefveld W (2000) Understanding the role of sink patches in source-sink metapopulations: reed warbler in an agricultural landscape. Conserv Biol 14: 1881–1892. 10.1111/j.1523-1739.2000.99022.x 35701934

[47] Vos CC, Verboom J, Opdam PFM, ter Braak CJF (2001) Toward ecologically scaled landscape indices. Am Nat 157: 24–41. 18707233 10.1086/317004

[48] Verboom J, Foppen R, Chardon P, Opdam P, Luttikhuizen P (2001) Introducing the key patch approach for habitat networks with persistent populations: an example for marshland birds. Biol Conserv 100: 89–101.

[49] Hanski I (1999) Metapopulation Ecology. Oxford, UK: Oxford University Press. 313 p.

[50] Hanski I (2001) Spatially realistic theory of metapopulation ecology. Naturwissenschaften 88: 372–381. 11688412 10.1007/s001140100246

[51] Schnell JK, Harris GM, Pimm SL, Russell GJ (2013) Estimating extinction risk with metapopulation models of large-scale fragmentation. Conserv Biol doi:10.1111/cobi.12047 10.1111/cobi.12047 23551595

[52] Hanski I, Ovaskainen O (2000) The metapopulation capacity of a fragmented landscape. Nature 404: 755–758. 10783887 10.1038/35008063

[53] Myers N, Mittermeier RA, Mittermeier CG, Da Fonseca GAB, Kent J (2000) Biodiversity hotspots for conservation priorities. Nature 403: 853–858. 10706275 10.1038/35002501

[54] da Fonseca GAB (1985) The vanishing Brazilian Atlantic forest. Biol Conserv 34: 17–34.

[55] Morellato LPC, Haddad CFB (2000) Introduction: the Brazilian Atlantic Forest. Biotropica 32: 786–792.

[56] Ribeiro MC, Metzger JP, Martensen AC, Ponzoni FJ, Hirota MM (2009) The Brazilian Atlantic Forest: how much is left, and how is the remaining forest distributed? Implications for conservation. Biol Conserv 142: 1141–1153.

[57] Brooks TM, Balmford A (1996) Atlantic forest extinctions. Nature 380: 115.

[58] Goerck JM (1997) Patterns of rarity in the birds of the Atlantic Forest of Brazil. Conserv Biol 11: 112–118.

[59] Ribon R, Simon JE, de Mattos GT (2003) Bird extinctions in Atlantic Forest fragments of the Viçosa Region, Southeastern Brazil. Conserv Biol 17: 1827–1839.

[60] Bogaert J, Zhou L, Tucker CJ, Myneni RB, Ceulemans R (2002) Evidence for a persistent and extensive greening trend in Eurasia inferred from satellite vegetation index data. J Geophys Res 107: 4119.

[61] Therivel R (2004) Strategic Environmental Assessment in Action. Earthscan. 276 p.

[62] Broadbent EN, Asner GP, Keller M, Knapp DE, Oliveira PJC, et al (2008) Forest fragmentation and edge effects from deforestation and selective logging in the Brazilian Amazon. Biol Conserv 141: 1745–1757.

[63] Hanski I (1994) A practical model of metapopulation dynamics. J Anim Ecol 63: 151–162.

[64] Hanski I (1998) Metapopulation dynamics. Nature 396: 41–49.

[65] Van Houtan KS, Pimm SL, Halley JM, Bierregaard Jr RO, Lovejoy TE (2007) Dispersal of Amazonian birds in continuous and fragmented forest. Ecol Lett 9: 1–11. 10.1111/j.1461-0248.2007.01004.x 17305805

[66] BirdLife International (2011) IUCN Red List for birds. Available: http://www.birdlife.org/. Accessed 2011 Aug.

[67] Harris GM, Pimm SL (2004) Bird species’ tolerance of secondary forest habitats and its effects on extinction. Conserv Biol 18: 1607–1616.

[68] Şekercioğlu ÇH (2007) Conservation ecology: area trumps mobility in fragment bird extinctions. Curr Biol 17: R283–R286. 17437705 10.1016/j.cub.2007.02.019

[69] Tufte ER (2001) The visual display of quantitative information. Cheshire, CT: Graphics Press. 197 p.

[70] Freckleton RP, Harvey PH, Pagel M (2002) Phylogenetic analysis and comparative data: a test and review of evidence. Am Nat 160(6): 712–726. 18707460 10.1086/343873

[71] Willis EO (1974) Populations and local extinctions of birds on Barro Colorado Island, Panama. Ecol Monogr 44: 153–169.

[72] Wilcove DS (1985) Nest predation in forest tracts and the decline of migratory songbirds. Ecology 66: 1211–1214.

[73] Leck CF (1979) Avian extinctions in an isolated tropical wet-forest preserve, Ecuador. Auk 96: 343–352.

[74] Clark CW, Rosenzweig ML (1994) Extinction and colonization processes: parameter estimates form sporadic surveys. Am Nat 143(4): 583–596.

[75] Russell GJ, Diamond JM, Pimm SL, Reed TM (1995) A century of turnover: community dynamics at three timescales. J Anim Ecol 64: 628–641.

[76] Sutherland GD, Harestad AS, Price K, Lertzman KP (2000) Scaling of natal dispersal distances in terrestrial birds and mammals. Conserv Ecol 4: 16.

[77] Bowman J (2003) Is dispersal distance of birds proportional to territory size? Can J Zool 81: 195–202.

[78] Hansbauer MM, Storch I, Leu S, Nieto-Holguin J-P, Pimentel RG, et al (2008) Movements of neotropical understory passerines affected by anthropogenic forest edges in the Brazilian Atlantic rainforest. Biol Conserv 141: 782–791.

[79] Hansbauer MM, Storch I, Pimentel RG, Metzger JP (2008) Comparative range use by three Atlantic Forest understorey bird species in relation to forest fragmentation. J Trop Ecol 24: 291–299.

[80] Marini MÂ (2010) Bird movement in a fragmented Atlantic Forest landscape. Stud Neotrop Fauna Environ 45: 1–10.

