# Supplementary material for: Quantitative Analysis of Forest Fragmentation in the Atlantic Forest Reveals More Threatened Bird Species than the Current Red List
Source: PLoS One. 2013 May 29;8(5):e65357. doi: 10.1371/journal.pone.0065357 (PMC3667042; doi:10.1371/journal.pone.0065357)
Supplement: Methods S1 — Spatially explicit metapopulation models and metapopulation capacity. (DOCX) [file pone.0065357.s001.docx]

**Methods S1**

**Spatially explicit metapopulation models and metapopulation capacity**

Levins (1969, 1970) first laid out metapopulation theory mathematically by defining the change in the fractional occupancy of a set of identical patches as:

$$\frac{dp}{dt}=cp(1-p)-ep$$

where *p* is the average fraction of patches occupied and *c* and *e* are constants measuring colonization and extinction respectively. The first step to making this model apply to heterogeneous patches arrayed in realistic space is to recast it as a set of equations of colonization and extinction rates, applying to each patch *i*:

$$col_{i}=c\sum\exp(-\alpha d_{ij})A_{j}p_{j}(t)$$

$$ext_{i}=\frac{e}{A_{i}}$$

Here, *p_j_* is the occupancy rate of patch *j*, *d_ij_* is the distance between patches *i* and *j*, 1/ α is the average migration distance, and *A_i_* and *A_j_* are the areas of patch *i* and *j* respectively. Hanski (1994) made the colonization and extinction terms dependent on patch areas and isolation:

$$\frac{dp_{i}}{dt}=c\sum_{j\neq i} f(D_{ij})A_{j}p_{j}(1-p_{i})-\frac{e}{A_{i}^{z}}p_{i}$$

where *e* and *c* are still constants, *z* describes how extinction rate varies with patch area (and is often about 0.5 for a wide range of taxa), *f(D_ij_)* is a function of the distance between patches *i* and *j* (for which we integrated Van Houtan’s (2007) heavy-tailed log-sech function — see below), and *p_j_* is still the occupancy rate of patch *j* (as only an occupied patch can provide colonists).

Realizing that the overall extinction rate, and especially the colonization rate, are not known for most species, Hanski & Ovaskainen (2002) took the spatially explicit components of the colonization term [*f(D_ij_)A_j_*] and divided them by the spatial component of the extinction term [1/ *A_i_*^x^] to create a matrix **M** with terms:

$$m_{ij}=\left\{\begin{matrix} f(D_{ij})A_{j}A_{i}^{z} & j\neq i \\ 0 & j=1 \end{matrix}\right.$$

The leading eigenvalue of matrix **M**, λ_M_, they termed the ‘metapopulation capacity.’ It is a measure of how the spatial configuration of a set of patches contributes to estimates of long-term metapopulation persistence. To overcome the non-intuitive behavior of this metric for systems of a few large patches, we added a self-colonization component (*m_ij_* = *A_j_A_i_* when *j* = *i*), effectively giving large patches the potential to harbor small numbers of survivors following an ‘extinction’ event. These survivors can recolonize the rest of the patch (Schnell et al. 2013):

$$m_{ij}=\left\{\begin{matrix} f(D_{ij})A_{j}A_{i}^{z} & j\neq i \\ A_{j}A_{i}^{z} & j=i \end{matrix}\right.$$

This measurement, metapopulation capacity with self-colonization, we use here for the analyses of the Atlantic forest bird ranges.

**The log-sech dispersal kernel and derived survival function**

Based on observed movements of understory birds though fragmented forest in the Amazon, Van Houtan at al. (2007) concluded that the dispersal kernel was best modeled by the heavy-tailed log-sech probability function. To convert this to a survival function we calculate the integral describing the right tail of the distribution (probability of moving that distance or further).

$$f(D_{ij})=\int\frac{2/(\pi br)}{(r/\alpha)^{1/b}+(r/\alpha)^{-1/b}}=\frac{2\tan^{-1}(a/D_{ij})^{\beta-1}}{\pi}$$

where α is a scaled measure of average dispersal distance (α > 0), β is the shape parameter of the kernel or β = 1 + 1/*b*.

1. Hanski I (1994) A practical model of metapopulation dynamics. J of Anim Ecol **63:** 151-162.
2. Hanski I, Ovaskainen O (2002) Extinction debt at extinction threshold. Conserv Biol **16:** 666-673.
3. Levins R (1969) Some demographic and genetic consequences of environmental heterogeneity for biological control. Bull Entomol Soc Am 15: 237-240.
4. Levins R (1970) Extinction. Pages 77-107 in M. Gesternhaber, editor. Some mathematical problems in biology. American Mathematical Society, Providence, Rhode Island.
5. Schnell JK, Harris GM, Pimm SL, Russell GJ (2013) Estimating extinction risk with metapopulation models of large-scale fragmentation. Conserv Ecol doi: 10.1111/cobi.12047
6. Van Houtan KS, Pimm SL, Halley JM, Bierregaard Jr RO, Lovejoy TE (2007) Dispersal of Amazonian birds in continuous and fragmented forest. Ecol Lett **9:** 1-11.
